# Valganciclovir is not associated with decreased EBV infection rate in pediatric kidney transplantation

**DOI:** 10.3389/fped.2022.1085101

**Published:** 2023-01-10

**Authors:** Elodie Cheyssac, Hamidou Savadogo, Nathan Lagoutte, Véronique Baudouin, Marina Charbit, Robert Novo, Anne-Laure Sellier-Leclerc, Marc Fila, Stéphane Decramer, Elodie Merieau, Ariane Zaloszyc, Jérôme Harambat, Gwenaelle Roussey

**Affiliations:** ^1^ Department of Pediatric Nephrology, Robert Debré University Hospital, APHP, Paris, France; ^2^ Department of Pediatrics, Pediatric Nephrology Unit, Nantes University Hospital, Nantes, France; ^3^ Department of Pediatric Nephrology, Necker Enfants Malades University Hospital, APHP, Paris, France; ^4^ Pediatric Nephrology Unit, Lille University Hospital, Lille, France; ^5^ Department of Pediatric Nephrology, HFME, Lyon University Hospital, Lyon, France; ^6^ Pediatric Nephrology Unit, Montpellier University Hospital, Montpellier, France; ^7^ Pediatric Nephrology Unit, Toulouse University Hospital, Toulouse, France; ^8^ Department of Pediatrics, Tours University Hospital, Tours, France; ^9^ Department of Pediatrics, Strasbourg University Hospital, France, Strasbourg, France; ^10^ Pediatric Nephrology Unit, Bordeaux University Hospital, Bordeaux, France

**Keywords:** prophylaxis, pediatric kidney transplantation, valganciclovir, Epstein–Barr virus, PTLD

## Abstract

**Introduction:**

Primary infection or reactivation of Epstein-Barr Virus (EBV) is a significant cause of morbidity and mortality in pediatric kidney transplantation. Valganciclovir (VGC) treatment is recommended for prophylaxis of cytomegalovirus infection, but its role for the prevention of EBV infection remains controversial.

**Patients and methods:**

All pediatric kidney transplant recipients aged <18 years old were considered for inclusion in this retrospective study. EBV negative recipients with an EBV positive donor (a group at risk of primary infection) or EBV positive recipients (a group at risk of reactivation) were included. Severe infection was defined by post-transplant lymphoproliferative disorder (PTLD), symptomatic EBV infection or by asymptomatic EBV infection with a viral load >4.5 log/ml. Outcomes were compared between patients receiving VGC prophylaxis (group P+) and those not receiving VGC prophylaxis (group P−).

**Results:**

A total of 79 patients were included, 57 (72%) in the P+ group and 22 (28%) in the P− group; 25 (31%) were at risk of primary infection and 54 (69%) at risk of reactivation. During the first year post-transplant, the occurrence of severe EBV infection was not different between the P+ group (*n* = 13, 22.8%) and the P− group (*n* = 5, 22.7%) (*p* = 0.99). Among patients at risk of primary infection, the rate of severe EBV infection was not different between the two groups (42.1% in P+ vs. 33.3% in P−). A higher frequency of neutropenia was found in the P+ group (66.6%) than in the P− group (33.4%) (*p* < 0.01).

**Conclusion:**

Our observational study suggests no effect of VGC for the prevention of EBV infection in pediatric kidney transplant recipients, irrespective of their EBV status. Adverse effects revealed an increased risk of neutropenia.

## Introduction

Kidney transplantation is the treatment of choice for kidney failure in children (1). However, the immunosuppressive therapy essential to prevent graft rejection is associated with infectious complications, especially viral infections (2, 3). Some of these infections can be transmitted by the donor and are responsible for significant morbidity and mortality in transplanted children (4). Epstein-Barr virus (EBV) is a herpes virus, affecting more than 95% of adults, and persists life-long in B lymphocytes (5, 6). After transplantation, EBV can be responsible for B cell proliferation and post-transplant lymphoproliferative disorder (PTLD) (7–9). The risk of PTLD is increased for EBV-negative recipients receiving a transplant from an EBV-positive donor (EBV mismatch), which is particularly observed in pediatric patients (9, 10). EBV mismatch may occur in about 30%–40% of pediatric kidney transplantations (10, 11). The value of antiviral prophylaxis in the prevention of EBV-related PTLD remains debatable, however some studies have suggested the efficacy of valganciclovir (VGC) which is widely used for the prevention of cytomegalovirus (CMV) infection (12, 13). We conducted a retrospective multicenter study in order to assess the potential role of VGC in the prevention of primary EBV infection or reactivation of EBV, in a French cohort of pediatric kidney transplant patients.

## Patients and methods

### Study population

This was a retrospective, multicenter, observational study including children under 18 years old who received a kidney transplant between January 2012 and June 2013. Ten out of the 12 French pediatric kidney transplant centers participated in the study (Bordeaux, Lille, Lyon, Montpellier, Nantes, Paris Necker, Paris Robert Debré, Strasbourg, Toulouse and Tours).

We included EBV-negative recipients (R−) at the time of transplantation who received a transplant from an EBV-positive donor (D+), and EBV-positive recipients (R+) at the time of transplantation regardless of donor EBV status. EBV-negative recipients who received a transplant from an EBV-negative donor (D−) were excluded because they were not considered to be at high risk for EBV severe infection during the 12 months of follow-up. Children who received a combined transplantation (liver-kidney, kidney-pancreas) and those whose follow-up was incomplete or died during the first year post-transplant were also excluded.

### Data collection and definitions

Data were collected from medical records by one investigator in each center using a standardized data collection form. Demographic, clinical and laboratory data were collected during the first year after the transplant. The timing of data collection during the first year post transplantation were twice a month in the first 3 months, then monthly until 12 month.

The indication for treatment by VGC depended on the protocol of each center (mostly for prevention of CMV infection), clinical practices were not modified for this study. VGC was administered for 6 months. Two groups of patients were defined: patients receiving VGC prophylaxis (P+) and patients without VGC prophylaxis (P−). Patients were considered to be at risk of EBV primary infection when they were EBV-negative and received a graft from an EBV-positive donor. They were at risk of EBV reactivation when they were EBV-positive at the time of transplantation.

VGC dose was calculated according to the previously published formula: dose (mg per day) = 7 × glomerular filtration rate (GFR) × body surface area (BSA) (14). GFR was estimated by the updated Schwartz formula (15) and BSA was estimated by the formula: [4 × weight (kg) + 7]/[weight (kg) + 90].

All centers used a technique of real-time PCR assays to measure EBV load in peripheral blood sample; but for “in house” or “homebrew” systems, primers and probes differed across laboratories. All values were converted into logarithmic unit.

In the sub-group at risk of primary infection, EBV infection was defined by a positive EBV PCR during the follow-up. In the subgroup at risk of reactivation, EBV infection was defined by an EBV PCR greater than 2.5 log/ml. Severe EBV infection was arbitrarily defined by an EBV PCR greater than 4.5 log/ml or by the presence of symptoms such as tonsillitis, lymphadenopathy, fever, hepatomegaly or splenomegaly, and lymphoproliferative syndrome with positive EBV PCR. Lymphoproliferative syndrome was defined by supra-centimetric lymphadenopathy, rapid increase in the volume of the tonsils, and tonsillar ulceration with a persistent EBV viral load above 4.5 log/ml.

The primary outcome was the incidence of severe EBV infection according to VGC treatment within the first year of kidney transplantation. Secondary outcomes were the incidence and timing of EBV infection across the cohort and subgroups (primary infection or reactivation), the presence of overimmunosuppression markers [defined as multiple warts >10, multiple molluscum contagiosum lesions >30, frequent ear/nose/throat (ENT) or respiratory infections, reactivation of other viruses], and the incidence of neutropenia.

### Statistical analyses and ethical issues

The characteristics of the two groups were compared using Chi-2, Fisher, Student or Wilcoxon tests according to the distribution of the variables. The percentage of severe EBV infections at 12 months was compared between the prophylaxis group (P+) and the group without prophylaxis (P−) using a Chi-2 test. EBV infection-free survival curves were estimated in the two groups by the Kaplan-Meier method and compared using a Log-Rank test. A *p* value <0.05 was considered statistically significant. Analyses were performed using SAS 9.4 software.

A study non-opposition form was collected from parents and inserted into patient records. The study was approved by the local ethics committee (registration number 14,285) from Nantes University Hospital.

## Results

### Characteristics of the population

**Figure 1:**
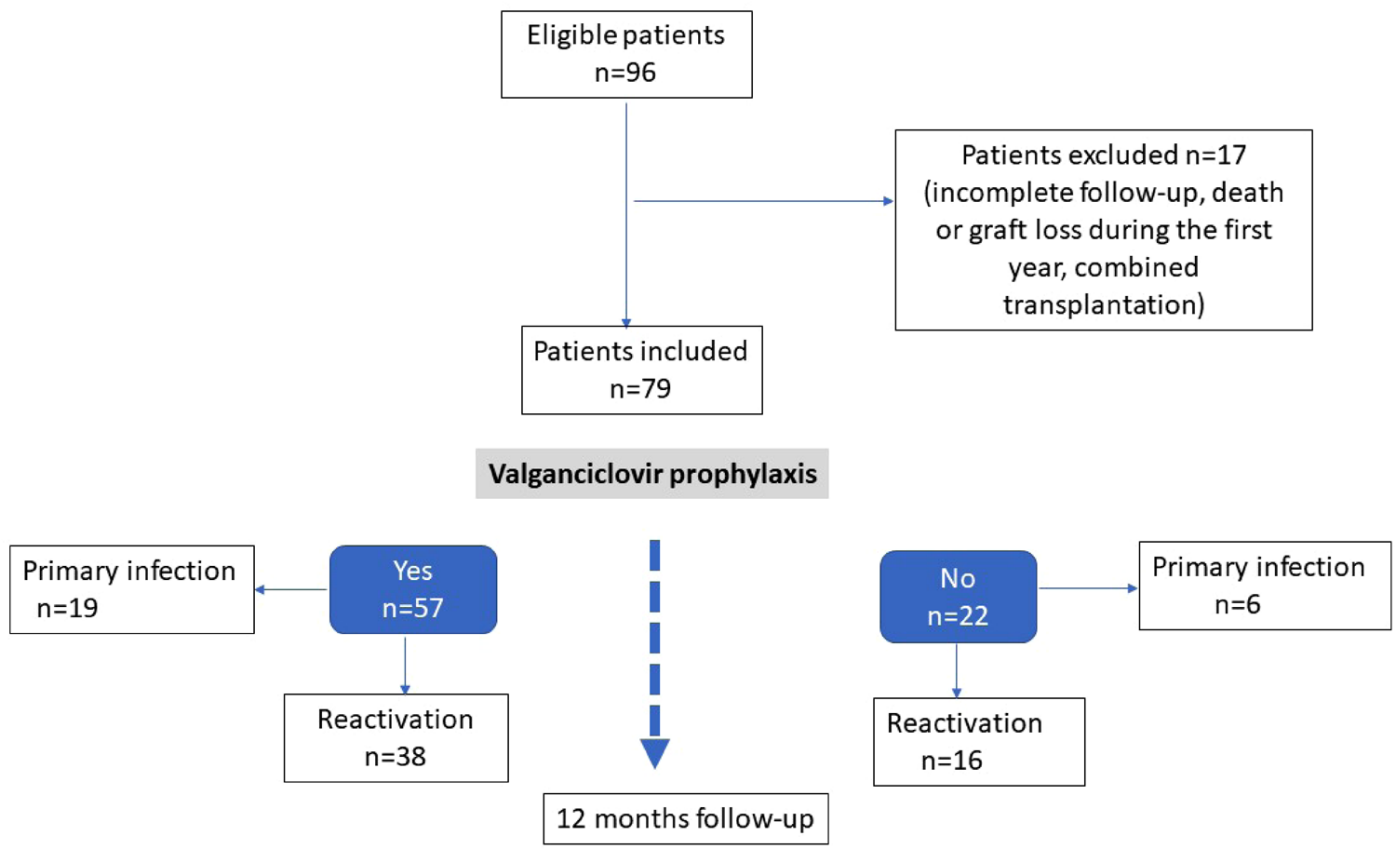
Flow chart of the study.

During the study period, 96 children underwent kidney transplantation in the participating centers. Seventeen patients were not included: 11 incomplete follow-up during the first year of transplantation, one death, one early graft loss, 3 combined liver-kidney transplant, 1 EBV-negative recipient with EBV-negative donor. A total of 79 patients were included, 57 (72%) in the prophylaxis group (P+) and 22 (28%) in the group without prophylaxis (P−). The two groups were comparable on all criteria apart from the donor/recipient CMV mismatch and the frequency of EBV viral load monitoring during follow-up (Table 1). The flow chart of the study is indicated in the Figure 1. As expected, a CMV mismatch (CMV donor positive/recipient negative) was more frequent in the P+ group (33% vs. 9%, *p* = 0.03). The mean number of EBV viral load performed was also higher in the P+ than in the P− group (11.5 vs. 7.9, *p* < 0.01).

**Table 1:** Patients characteristics.

Variables	No prophylaxis (*n* = 22)	Prophylaxis (*n* = 57)	*p*
Age at transplantation (mean ± SD)	12.0 (±3.7)	12.4 (±4.4)	0.48
Male sex (%)	12 (54.6%)	33 (57.9%)	0.64
Primary kidney disease (%)			0.71
CAKUT	8 (36.4%)	18 (31.6%)	
Acquired glomerulopathy	4 (18.2%)	16 (28.0%)	
Hereditary nephropathy	5 (22.7%)	14 (24.5%)	
Vascular nephropathy	1 (4.5%)	2 (3.5%)	
Others	4 (18.2%)	6 (10.5%)	
Deceased donor	17 (77.3%)	49 (85.9%)	0.26
Number of EBV viral load monitoring during the first year	7.9	11.5	0.001
EBV status at transplantation			0.60
Primary infection risk (D+/R−)	6 (27.3%)	19 (33.3%)	
Reactivation risk (R+)	16 (72.7%)	38 (66.7%)	
CMV status at transplantation			
CMV Negative	13 (59.1%)	32 (56.1%)	0.81
CMV mismatch (D+/R−)	2 (9.1%)	19 (33.3%)	0.03
HLA A-B-DR mismatches	3.3 (±0.9)	3.4 (±1.0)	0.70
Induction therapy			1.00
Anti-IL2-Receptor antibody	18 (81.8%)	47 (82.4%)	
Antithymocyte globulin	4 (18.2%)	9 (15.8%)	
Initial immunosuppressive therapy			0.60
Tacrolimus	17 (77.3%)	40 (70.2%)	
Ciclosporine	5 (22.7%)	16 (28.1%)	
MMF	17 (77.3%)	52 (91.2%)	
Azathioprine	4 (18.2%)	4 (7.0%)	
Corticosteroids	22 (100%)	51 (89.4%)	

### Severe EBV infection

**Figure 2:**
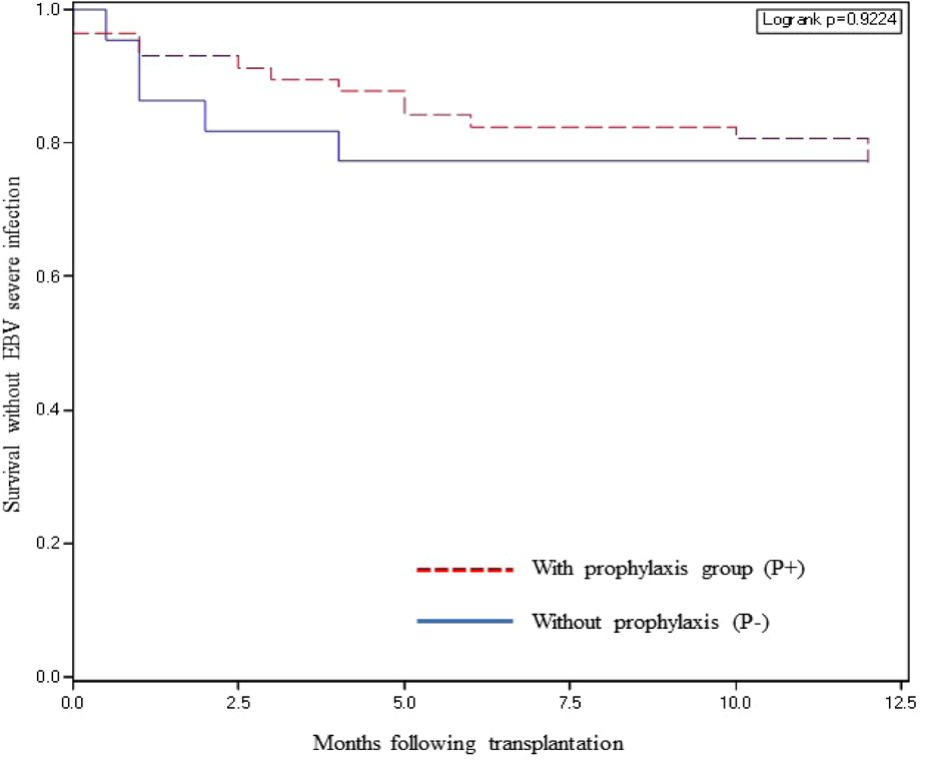
Survival without severe EBV infection according to valganciclovir prophylaxis.

Thirteen patients (22.8%) in the P+ group and 5 (22.7%) the P− group presented a severe EBV infection within one year of transplantation (*p* = 0.99). The mean time to severe EBV infection was longer in the P+ than in the P− group (4.7 ± 4.2 months vs. 1.7 ± 1.4 months). Survival without severe EBV infection is illustrated in the Figure 2. In the P+ group, 2 out of 57 patients (3.5%) developed a histologically proven PTLD during the first year of transplantation while no patient from the P− group had a PTLD.

### EBV infection

**Figure 3:**
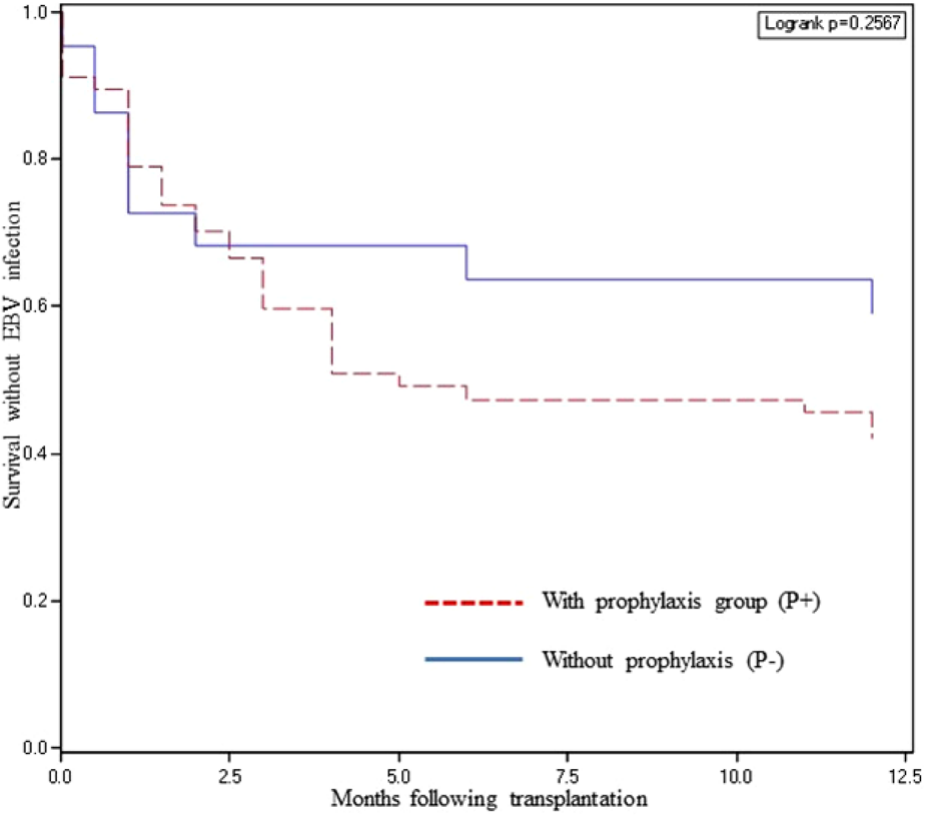
Survival without EBV infection according to valganciclovir prophylaxis.

There was no significant difference in the incidence of EBV infection in the P+ and P− groups (57.9% vs. 40.9% respectively, *p* = 0.17). The mean time to EBV infection was similar in the two groups (3.0 ± 3.2 months in P+ vs. 2.7 ± 3.9 months in P−). The survival without EBV infection was not significantly different between the P+ and P− groups (*p* = 0.26, Figure 3).

### Subgroup at risk of primary EBV infection

Among patients at risk of primary EBV infection, 8 out of 19 patients (42.1%) who received VGC prophylaxis experienced a severe EBV infection, compared to 2 out of 6 patients (33.3%) without prophylaxis. There was no significant difference in the incidence of severe EBV infection at one year post-transplantation (*p* = 0.94).

### Sub group at risk of EBV reactivation

Among patients at risk of EBV reactivation, there was no difference in the rate of severe EBV infection between patients who received VGC prophylaxis (13.1%) and those who did not (18.7%). Survival without a severe EBV infection at one year was not significantly different (*p* = 0.61).

### Factors associated with severe EBV infection

No demographical or clinical factor (age, sex, HLA mismatch) was associated with the occurrence of a severe EBV infection (Table 2). Anti-thymocyte globulin induction was associated with an increased rate of a severe EBV infection (7 out 13 patients receiving ATG, 53%) when compared to an induction with basiliximab (*n* = 11 out 66 patients receiving basiliximab, 16%) (*p* = 0.008). The maintenance treatment with tacrolimus or ciclosporine, mycophenolate mofetil or azathioprine, did not influence the risk of severe EBV infection. The area under the curve (AUC) of mycophenolic acid (MPA) after three months was significantly lower in patients with severe infection (*p* = 0.01). Among patients with a severe EBV infection, 55.5% exhibited some markers of overimmunosuppression compared to 41.0% in those without severe EBV infection (*p* = 0.23). The risk of neutropenia was significantly increased in the P+ group (66.6%) compared to the P− group (31.8%, *p* = 0.005).

**Table 2:** Patients with or without EBV infection according to associated risk factors.

Variables	EBV infection (*n* = 42)	No EBV infection (*n* = 37)	*p*	Severe EBV infection (*n* = 18)	No severe EBV infection (*n* = 61)	*p*
Male sex	20 (49%)	26 (69%)	0.07	10 (56%)	36 (59%)	0.82
Age at transplantation (years)	12.2 (±4.2)	12.4 (±4.2)	0.83	11.1 (±4.7)	12.7 (±4.0)	0.20
HLA A-B-DR mismatches	4.3 (±1.3)	4.4 (±1.1)	0.73	4.2 (±1.4)	4.3 (±1.2)	0.81
Presence of HLA DR7	8 (20%)	14 (39%)	0.07	5 (28%)	18 (29%)	0.90
Induction therapy			0.48			0.008
Anti-IL2-Receptor antibody	34 (81%)	32 (86%)		11 (61%)	55 (90%)	
Antithymocyte globulin	8 (19%)	5 (14%)		7 (39%)	6 (10%)	
Maintenance treatment						
Tacrolimus	32 (76%)	26 (70%)	0.60	13 (72%)	45 (73%)	1
Ciclosporine	10 (24%)	11 (30%)	0.60	5 (28%)	16 (27%)	1
MMF	36 (85%)	34 (92%)	0.49	15 (83%)	55 (90%)	0.42
Azathioprine	5 (12%)	3 (8%)	0.71	2 (11%)	6 (10%)	1
Corticosteroids	37 (88%)	37 (100%)	0.05	14 (78%)	36 (59%)	0.01
AUC MMF at month 3	48.2 (±19.3)	56.5 (±20.9)	0.34	33.9 (±19.5)	55.8 (±18.6)	0.01
Mean tacrolimus trough level during 12 months	5.6 (±4.0)	6.0 (±4.1)	0.57	4.4 (±3.4)	6.2 (±4.1)	0.27
CMV infection	15 (36%)	9 (24%)	0.27	5 (28%)	19 (31%)	0.78
Presence of immunosuppression markers	23 (55%)	13 (36%)	0.01	11 (61%)	26 (43%)	0.23
eGFR at 12 months (ml/min/1.73 m^2^)	72.9 (±19.6)	68.2 (±21.2)	0.16	76.5 (±25.2)	69.1 (±18.8)	0.16
Rejection	8 (19%)	11 (30%)	0.27	3 (17%)	16 (26%)	0.54

## Discussion

This study compares the occurrence of EBV infection with or without VGC prophylaxis after kidney transplantation in children. We found that VGC prophylaxis was not associated with a lower risk of severe EBV infection in the first year after kidney transplantation in pediatric patients. VGC did not seem to be effective, even in the high-risk group, when the donor was EBV-positive and the recipient was EBV-negative. Additionally, VGC did not seem to have any influence on the reactivation of EBV during the first year after transplantation among EBV-positive recipients. However, side effects such as neutropenia possibly related to VCV were more common in patients who received treatment with VGC.

Viral infections are common complications occurring after kidney transplantation (16–18). EBV is a major challenge in organ transplantation because it can lead to an uncontrolled proliferation of B cells, known as PTLD, both in children and adults. In the majority of cases, PTLD is associated with active replication of EBV so that negative EBV serology at the time of transplantation and EBV infection are major risk factors for early PTLD (8, 9, 19, 20). This can lead to a decrease or cessation of immunosuppression with the risk of rejection and graft loss.

The known risk factors of PTLD are an unfavorable EBV mismatch (EBV D+/R−) with a high risk of EBV primary infection during the first months after transplant, young age, the use of antithymocyte globulin and increased tacrolimus trough levels (8, 10, 19–22).

The role of antiviral prophylaxis in the prevention of PTLD remains controversial (23). There are some hints on beneficial effects in some retrospective and prospective studies (10) despite the lack of significant difference between patients receiving antiviral prophylaxis and those who did not in a meta-analysis of solid organ transplant recipients (23). Our results differ from those of other authors who reported a possible effect of antiviral prophylaxis. Höcker et al. studied 28 pediatric kidney transplant recipients, 20 of whom received prophylaxis with VGC or ganciclovir. At the end of the one-year follow-up period, 45% of children under prophylaxis had a primary EBV infection vs. 100% of children without prophylaxis. Antiviral prophylaxis was associated with a significant decrease in EBV viral load (10). A study by Albatati et al. showed that VGC delayed the onset of EBV viremia in pediatric heart and kidney recipients (24). Darenkov et al. reported only one case of PTLD out of 198 adult recipients (0.5%) who received antiviral prophylaxis with acyclovir or ganciclovir (25). In pediatric liver transplantation, McDiarmid et al. compared two groups of patients: 18 in a high-risk group (EBV-D+/R−) and 22 in a low-risk group (D+/R+; D−/R−; D−/R+) (26). In the high-risk group, all patients received a minimum of 100 days of intravenous ganciclovir while, in the low-risk group, patients received intravenous ganciclovir during their initial hospitalization and then oral prophylaxis. No case of PTLD was noted in the high-risk group while two patients developed PTLD in the low-risk group (26). Malouf et al. reported a significant reduction of the incidence of PTLD in lung transplantation with antiviral prophylaxis in EBV-seronegative patients (27). Funch et al. showed that the administration of an antiviral agent (acyclovir or ganciclovir) reduced the risk of presenting a PTLD by up to 83% when they compared adults and children with a PTLD to the rest of the cohort, in particular in the first year after transplantation (28). European Best Practice Guidelines for kidney transplantation in 2002 recommend antiviral prophylaxis by acyclovir, valacyclovir or valganciclovir in patients at high-risk of primary EBV infection, starting at the time of the transplant and lasting for at least three months (29). Yager et al. reported in a randomized, double-blind, placebo-controlled study, a reduction in EBV replication (oral excretion) in a sample of 26 adults who received VGC for 8 weeks (30).

Consistent with our results, other authors showed that antiviral prophylaxis did not appear to be effective in preventing EBV infections in pediatric kidney transplantation. Paulsen et al. found a high frequency of EBV infections (67%) in a retrospective cohort of 92 children who received a kidney transplant and were treated with VGC prophylaxis (31). Yamada et al. found that antiviral drugs were not effective in preventing EBV infections, nor decreasing EBV viral load in their cohort of pediatric kidney transplant recipients (32). A meta-analysis of 31 studies evaluated the effect of antiviral prophylaxis on the onset of PTLD in EBV-naïve recipients who had received a solid organ transplant from an EBV-positive donor. This meta-analysis did not find a statistically significant difference between patients receiving antiviral prophylaxis (VGC or other) and those who did not receive prophylaxis (23). The authors concluded that antiviral prophylaxis in patients at high risk of EBV primary infection in solid organ transplantation has no effect on the occurrence of PTLD (23). Of note, in our study, the 2 patients who developed a biopsy proven PTLD were in the prophylaxis group. Another meta-analysis published in 2018 suggested that there is no advantage in the use of antiviral drugs for PTLD prophylaxis (33). Other strategies could be explored, especially rituximab as a pre-emptive treatment (34).

EBV infection may be subclinical and cause chronic graft injury (35). Smith et al. found a subclinical EBV infection of 36% in 55 pediatric recipients during the first 2 years of kidney transplantation. Virological surveillance by DNA PCR allows early diagnosis of these asymptomatic EBV infections (35, 36). Our study also aimed to identify other factors associated with the occurrence of EBV infections. The type of induction therapy may influence EBV infections. Therefore, anti-thymocyte globulin which leads to greater and longer-lasting immunosuppression, was associated with an increased risk of severe EBV infection. However, the number of patients who received anti-thymocyte globulin is small in our cohort. According to a recent review (37), the optimal induction therapy remains controversial, and the choice of immunosuppressive drugs must take into account the characteristics of the patient. No clear role of steroids on development of EBV infection can be suggested based on our data. The AUC of MPA was significantly lower in patients with severe EBV infection in our study. This could be explained by the voluntary decrease in immunosuppression before AUC in these patients to overcome from EBV infection. However one could oppose the inhibitory effect of MPA on the proliferation of EBV-infected B lymphocytes (38). Smith et al. and Li et al. showed that EBV infection, even subclinical, was associated with chronic graft damage and decreased graft function in pediatric recipients (35, 39). However, in our study, impaired graft function (assessed by mean eGFR) was observed in the group of decreased EBV infections. This may be explained by a voluntary reduction in the dose of anticalcineurin, to limit the risk of over-immunosuppression in patients with a high EBV viral load, which may have led to reduced nephrotoxicity and increase in eGFR. It is noteworthy that the HLA DR7 allele appeared to be a risk factor of PTLD in two previous studies (18, 40), however this was not confirmed in our study.

VGC prophylaxis was associated with a significant increase in the occurrence of neutropenia. This may be explained by the myelotoxicity of VGC although neutropenia may rely on a completely different reason than only the administration (or non-administration) of a VCG prophylaxis. Neutropenia increases the risk of infection and may lead to discontinue other neutropenic treatments such as mycophenolate mofetil or cotrimoxazole, thus increasing the risk of rejection or pneumocystis (41). Finally, we did not investigate the association between administered VGC dosage and the incidence of EBV infections. Some centers perform pharmacokinetic determinations of VGC during follow-up in order to adjust dosages to the AUC and to limit treatment complications. A recent study in adult patients suggests that this practice tends to decrease the time to clear CMV, and that hematological complications may be related to overexposure to VGC (42).

Our study has several limitations. It was conducted retrospectively with a monitoring protocol that was intended to be common. However, the number of EBV viral load performed differed widely between centers. Therefore, one can speculate that some infections were underdiagnosed because of infrequent EBV PCR monitoring, especially in the group of patients who did not receive VGC prophylaxis. Indeed, one of the criteria for the severity of EBV infection was based on the quantification of the viral load. Patients who did not receive prophylaxis had statistically less frequent EBV viral load monitoring during the follow-up. It is possible that this difference in monitoring may have led to a decreased incidence of diagnosed infections in the P− group. Another limitation of the study is the definition of a severe EBV infection, we defined a value of greater than 4.5 log/ml EBV-DNA as indicative. However, there is no clear threshold value for a predictive use of EBV DNA concentrations, possible highly variable measurements between laboratories and often lacking use of international EBV standards (e.g., the first WHO standard from 2011) allowing a conversion of genome equivalents per ml into international units per ml. Although it was a multicenter study, it was a small population which is likely underpowered to detect differences between the groups. Moreover, data on BK viremia were not collected, such that its impact on immunosuppressive therapy, and on the development or severity of EBV infection could not be studied.

In conclusion, in this retrospective French multicentric cohort of pediatric kidney transplant recipients, VGC prophylaxis did not effectively prevent EBV infections, whether severe or not. However, this prophylaxis is statistically associated with the occurrence of neutropenia. Larger prospective cohort studies or clinical trials may provide more robust conclusions, and possibly recommendations for the prevention of EBV infections in pediatric kidney transplantation.

## Data Availability

The raw data supporting the conclusions of this article will be made available by the authors, without undue reservation.
